# Effects of an exclusive breastfeeding intervention for six months on growth patterns of 4–5 year old children in Uganda: the cluster-randomised PROMISE EBF trial

**DOI:** 10.1186/s12889-016-3234-3

**Published:** 2016-07-12

**Authors:** Lars T. Fadnes, Victoria Nankabirwa, Ingunn M. Engebretsen, Halvor Sommerfelt, Nancy Birungi, Carl Lombard, Sonja Swanevelder, Jan Van den Broeck, Thorkild Tylleskär, James K. Tumwine, for the PROMISE-EBF Study Group

**Affiliations:** Centre for International Health, Department of Global Public Health and Primary Care, University of Bergen, Post box 7804, 5020 Bergen, Norway; Department of Clinical Dentistry, University of Bergen, Bergen, Norway; Department of Paediatrics and Child Health, Makerere University, Kampala, Uganda; Centre for Intervention Science in Maternal and Child Health (CISMAC), University of Bergen, Bergen, Norway; Department of International Public Health, Norwegian Institute of Public Health, Oslo, Norway; Medical Research Council, Cape Town, Francie Van Zyl Drive, 7535 South Africa

**Keywords:** Anthropometry, Growth, Exclusive breastfeeding, Peer-counselling, Uganda

## Abstract

**Background:**

Breastfeeding promotion is regarded as one of the most effective interventions to improve child health, and could reduce under-5-mortality by 8 % globally. Few studies have assessed the health outcomes beyond infancy of interventions promoting exclusive breastfeeding.

**Methods:**

This study assessed growth in under-five children who participated in a cluster-randomised trial in Eastern Uganda (ClinicalTrials.gov.no.NCT00397150). In the intervention arm, peer counsellors promoted exclusive breastfeeding during the first 6 months of infancy. There were no interventions after 6 months of age. Mother-infant pairs were interviewed at visits scheduled at 3, 6, 12 and 24 weeks after birth and follow-up visits at 2 and 5 years, with 765 included in the analyses.

**Results:**

The mean length/height-for-age and weight-for-age-z-score (HAZ, WAZ) decreased with increasing age in both the intervention and control arms. At the three weeks visit, HAZ in the intervention was −0.45 (−0.68;−0.21) and −0.32 (−0.56;−0.07) in the control arm. At the 2 year follow-up, the mean HAZ in the intervention was −1.85 (95 % CI −1.97;−1.73) compared to −1.61 (−1.87;−1.34) in the control. Similarly, at the 5 year follow-up, the mean HAZ in the intervention was −1.78 (−2.08;−1.47) compared to −1.53 (−1.79;−1.28) in the control arm. At the 2 year follow-up visit, 139 (45 %) were stunted (HAZ<−2) in the intervention compared to 109 (37 %) in the control arm, odds ratio (OR) 1.7 (1.1;2.4). Underweight (WAZ<−2) was also more common in the intervention arm than in the control at the five years follow-up (OR 1.7 (1.0;2.8)), with a mean WAZ of −1.28 (−1.47;−1.08) and −1.06 (−1.19;−0.92) in the intervention and control arm, respectively.

**Conclusion:**

While stunting was widespread at 2 and 5 years of age in both arms, it was more common in the intervention arm. It is questionable whether community-based support from lay people with short training and focussing only on exclusive breastfeeding, is an appropriate strategy to improve child health and development.

**Trial registration:**

ClinicalTrials.gov.no.NCT00397150. Registered 7th of November 2006.

**Electronic supplementary material:**

The online version of this article (doi:10.1186/s12889-016-3234-3) contains supplementary material, which is available to authorized users.

## Background

Promotion of exclusive breastfeeding has been estimated to reduce under-5-mortality by 8 % [1, 2]. Numerous studies have assessed the effect on behaviour change of interventions to promote breastfeeding with either professional support or support from lay people [3]. A Cochrane review of 34 trials conclude that it is feasible to change breastfeeding practices with additional professional support – both to increase the duration of exclusive breastfeeding and breastfeeding duration in general [3].

We have earlier reported behavioural practice findings from the PROMISE-EBF trial in Uganda with an intervention with peer-counsellors to promote exclusive breastfeeding for 6 months. The intervention increased the proportion of participants practising exclusive breastfeeding [4]. At 12 weeks age based on 7-day recall, 77 % practiced exclusive breastfeeding in the intervention arm compared to 34 % in the control arm, while at 24 weeks 51 % practiced exclusive breastfeeding in the intervention and 11 % in the control arm.

One concern with exclusive breastfeeding promotion interventions has been negative growth outcomes especially in populations with widespread undernutrition [5], and a Cochrane review by Kramer and Kakuma recommended large randomised trials to rule out modest differences in risk of undernutrition of exclusive breastfeeding for 6 months [6]. A review by Giugliani and Victora reported that few studies have assessed the health outcomes of breastfeeding promotion – with an non-significant trend to better weight gains among those receiving breastfeeding support at 4 months [7]. To our knowledge, only two studies including the MINIMat trial in Bangladesh and the facility-based PROBIT-trial in Belarus have assessed growth outcomes after 1 year of age [8, 9]. These trials did not find any beneficial effect at the age of 4.5 and 6.5 years among children among those having been exclusively breastfed for a slightly longer duration.

To our knowledge, no African trials have reported on long term effects on child growth from breastfeeding support. We present growth outcomes from a 5 year follow-up of the PROMISE-EBF trial in Uganda, which was a community-based cluster-randomised controlled trial promoting exclusive breastfeeding for 6 months.

## Methods

This study assessed anthropometric outcomes from 5 years of follow-up of the PROMISE-EBF trial (ClinicalTrials.gov.no. NCT00397150) in Mbale District, Eastern Uganda [4, 10]. The study was conducted between 2006 and 2011. The PROMISE-EBF trial with 6 months of follow-up was also conducted in Burkina Faso and South Africa [4, 10].

### Study settings

Mbale had a population of 403,100 in 2008 (http://www.ubos.org/), and is predominantly rural with 59 % home deliveries. The first-visit attendance for antenatal care was 95 %. The under-5-mortality rate in 2004–5 was 137 per 1000 live births, and the regional HIV-prevalence among women was 6.2 % (http://www.ubos.org/, [11]).

### Study design

**Fig. 1:**
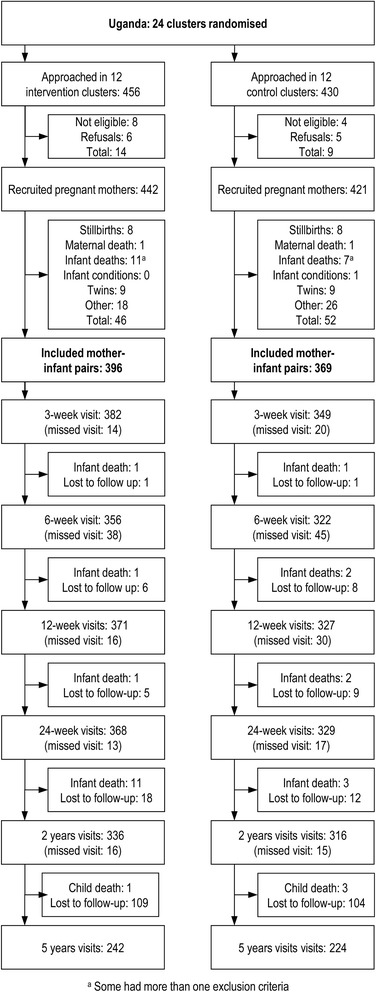
Trial profile overview

The sample size calculation was done to detect differences in infant feeding patterns and diarrhoea morbidity, and has been described elsewhere with a detailed description of the trial [4]. A total of 24 clusters within a 1 h drive from Mbale Municipality in Mbale District were chosen with a population corresponding to a birth rate of 35 per cluster. Each cluster had access to basic amenities such as water source, primary school, market or trading centre – independent of other clusters. The clusters were stratified on urban vs. rural when being randomised to intervention or control. From these clusters, 886 pregnant women (7 months or visibly pregnant) were approached with consecutive sampling of women who intended to breastfeed and remain in the cluster for the coming year, and 863 were recruited, Fig. 1. Of these, 98 were excluded due to death of infant or mother before 3 weeks after delivery, mothers having moved or being lost-to-follow-up, twin delivery, or conditions including severe malformations. Thus, 765 (89 %) mother-infant pairs remained in the analysis. The mother-infant pairs were interviewed at visits scheduled at 3, 6, 12 and 24 weeks after birth, with follow-up visits at around 2 and 5 years of age. The following time ranges were regarded as timely interview visits: 3: 1.5–4.5; 6: 4.5–9; 12: 9–18; 24: 18–28 weeks, 2 years: 1–3 years and 5 years: 3.5–5.5 years. Anthropometric measurements collected outside these ranges are not presented in the tables and figures. The median follow-up time for all included participants was 4.0 years (inter-quartile range [IQR] 1.8–4.7), and similarly 4.5 years (4.1–5.1) at the last follow-up visit.

**Fig. 2:**
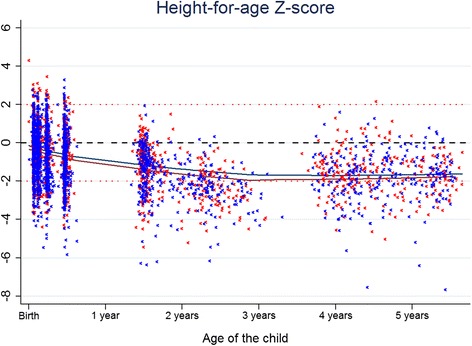
Distribution of height-for-age z-scores (HAZ) at up to 5 years of age presented as scatter plot with locally weighted regression smoothing (lowess). 1. *Blue* indicates those who were in the intervention arm and *red* those in the control arm (markers for each individual value and line indicating the smoothed regression lines). 2. The *black dashed horizontal line* represents the mean in the WHO growth standard reference population (HAZ = 0). 3. Those below the lower *red dotted horizontal line* were stunted (HAZ<−2)

The control clusters received no interventions and could use standard health care from the public health services. The standard health counselling involved some infant feeding counselling of varying quality usually taking place in antenatal clinics in public health facilities [12]. Mothers in the intervention clusters received breastfeeding support by peer-counsellors. These counsellors were trained in a 1 week course by a research team using a curriculum based on the WHO courses ‘*Breastfeeding Counselling: a Training Course*’ and ‘*HIV & Infant Feeding Counselling: a Training Course*’ [13, 14]. After training they returned to their communities and started supporting women planning to breastfeed and breastfeeding women within their respective geographical cluster while being followed up and supported in their work for 1 year [15]. Each mother was offered at least five visits with the first visit during pregnancy, and then subsequently scheduled at week 1, 4, 7 and 10 after delivery.

The peer counsellors provided information and supported EBF for 6 months. The peer counsellors identified common breastfeeding problems such as a feeling of having insufficient breast milk, sore nipples, breast engorgement, mastitis and poor positioning at the breast. The information they gave focused on good attachment and positioning, frequent breastfeeding, benefits of emptying one breast before changing to another breast, how to deal with a crying baby, expressing and storing breast milk, and how to assess baby stools and urination. The mothers who had any breastfeeding problems that could not be dealt with by the peer-counsellor were referred to a health worker with training in breastfeeding management.

Anthropometric measurements were carried out in line with the guidelines from WHO with the use of ‘*Baby/infant/adult Length-height measuring system SET 2*’ and ‘*Infant scale spring type, 25 kg, 100 g*’ from the UNICEF supplies [16]. Length was measured in the recumbent position to the nearest 0.1 cm and child weight was recorded to the nearest 0.1 kg. For the 5 year follow-up the height was measured in standing position using the same tools. The measures were taken by trained data collectors who were unaware of the trial arm allocation. Validity and reproducibility exercises were conducted at least twice annually during the data collection period. To assess anthropometric infant growth, we used the WHO growth standards as reference [17]. To calculate the Z-scores, the programs WHO Anthro and WHO AnthroPlus 1.0.4 were used.

### Data management

Data was electronically collected by data collectors speaking the local language Lumasaaba, and entered directly into handheld computers with the program EpiHandy using an electronic questionnaire [18]. For the follow-up visits data collection was done on paper and double entered into EpiData 3.1. Stata SE11 was used for statistical analysis (www.stata.com).

### Data cleaning

Data cleaning of the anthropometric data was done in two stages. First, the absolute differences for length and weight between measurements were checked for extreme outliers considered to be highly implausible. The second step was based on the attained z-scores from the WHO Child Growth Standards (with weight-for-age z-scores [WAZ], length/height-for-age z-scores [HAZ], weight-for-length/height z-scores [WLZ]). Measurements were regarded as implausible if:

1. WAZ <−6 or > 6;
2. HAZ <−6 or > 6;
3. WLZ <−5 or > 5 or
4. WLZ > 3 and HAZ <−3.

Similarly, extreme changes between two visits were regarded as implausible. This included when the difference in HAZ- and WLZ-scores between one interview and the prior and subsequent interview was more than 2.5 or 3 (between the first four visits during infancy). Unless explanations for implausible measurements were found (e.g. a note indicating marasmus), the measurements were set to missing. Around 6 % of the measurements were regarded as implausible and set to missing, mostly from the 3 week interview.

### Analysis

Categorical outcomes were analysed with a binomial generalised linear model while continuous outcomes were analysed with linear regression. Means were also calculated for anthropometric measurements. All analyses were adjusted for cluster and site (urban or rural) and the significance level was set to *p* <0.05.

There were some missing data mainly due to missed interview visits when caretakers were unavailable for two to three home visits (Fig. 1). The proportion of missing information was highest in the 5 year follow-up visit mainly due to relocation, and at the 3 week visit as many mothers stayed with their mothers or mothers-in-law for a period of time after birth. Baseline characteristics were compared between participants having valid and missing information to assess whether there were differences in missing (Additional file 1: Table S1). The proportion with missing anthropometric measurements were similarly distributed between the intervention and the control arms with the exception of the 12 weeks interview, where 50 (14 %) were missing in the control arm and 33 (8 %) in the intervention arm. A complete-subject analysis is only recommended when measurements are missing randomly. As all factors were not distributed completely symmetrically between the arms, analyses with an inverse-probability weighted method were carried out. A probit regression was used to calculate population weights based on the following factors: intervention or control arm allocation, likelihood of non-participation in the study based on missing measurements at other time points, site, socio-economic status, mother’s education and age, parity, gender of the infant, marital status, whether the child was weighted at birth, place of delivery, intended feeding strategy before delivery, and feeding practices at 12 and 24 weeks. The models gave more weight to cases with valid data that had characteristics associated with highest probability of having missing data. The means for the population weights in the different interviews were in the range from 1.1 to 1.2 for the first five visits and 1.7 for the 5 year follow-up.

A longitudinal analysis was done for time dependent change in WLZ, HAZ and WAZ to compare each arm, using a mixed model adjusting for both clustering and site at the respective visits. Linear prediction lines for each arm are presented. Change in the prevalence of stunting (HAZ<−2) over time was checked with a multilevel mixed-effects logistic regression.

Multiple correspondence analysis was used to construct a wealth index for the assessment of socio-economy based on ownership of assets and house characteristics including toilet facilities, number of rooms and beds, roof material, lantern, radio, television, bicycle and motor vehicles. This is analogous to using principal component analysis with categorical data [19]. The families of the children were grouped into quintiles on the basis of socio-economic rank.

## Results

The median age of the mothers at the time of recruitment was 25 years (IQR 20–30), with a median of 6 years of schooling (IQR4–8), and a body mass index of 21.8 (IQR 20.4–23.7), Table 1. Most lived in a rural area (566 [74 %]) and nearly all were married or cohabiting (701 [92 %]). Among the children, 387 (51 %) were males and 579 (77 %) had siblings.

**Table 1:** Background characteristics of the populations included in the trial and at the 2 and 5 years follow-up

Visit	Baseline (0 to 6 months)				2 years visit				5 years visit			
	Control		Intervention		Control		Intervention		Control		Intervention	
Categorical data	*n*	%	*n*	%	*n*	%	*n*	%	*n*	%	*n*	%
Eligible mother-infant pairs	369		396		297		310		221		242	
Multipara (child has siblings)	273	76	306	78	223	76	245	80	174	79	196	82
Previous child death	80	29	109	36	65	29	87	36	56	32	73	37
Female child	181	49	194	49	147	49	150	48	110	50	119	49
Mother married or cohabiting	338	92	363	93	274	93	284	92	209	95	224	93
Living in rural area	276	75	290	73	232	78	240	77	172	78	187	77
Electricity in house	70	19	53	14	52	18	40	13	37	17	30	13
Continuous data	Median	IQR	Median	IQR	Median	IQR	Median	IQR	Median	IQR	Median	IQR
Maternal age	24	(20–30)	25	(20–30)	24	(20–30)	25	(21–30)	25	(21–31)	26	(21–30)
Maternal education	6	(5–9)	6	(4–8)	6	(4–9)	6	(4–8)	6	(4–9)	6	(4–7)
Child age	(4 visits)		(4 visits)		19	(18–27)	19	(18–27)	53	(49–61)	55	(50–62)
Maternal body mass index	22	(20–24)	22	(20–24)	22	(21–24)	22	(20–24)	22	(21–24)	22	(20–24)
Socio-economic quintile	3	(2–4)	3	(2–4)	3	(2–4)	3	(2–4)	3	(2–4)	3	(2–4)

WLZ were lower in the intervention arm compared to the control in all interviews, but only statistically significantly so at 24 weeks, Additional file 1: Table S2. The HAZ and WAZ values were higher in the control arm than in the intervention arm in all visits, and WAZ was significantly different at 24 weeks, Tables 2 and 3. The distribution of the HAZ indicated that there was a clear left-shift away from the WHO growth standards, Additional file 1: Figure S1. The standard deviations of the anthropometric measurements at 3 weeks were 1.23 for HAZ and 1.10 for WAZ, at the 2 year follow-up they were 1.25 for HAZ and 1.19 for WAZ and at the 5 year follow-up 1.25 for HAZ and 1.05 for WAZ.

**Table 2:** Length/height-for-age z-scores (HAZ) with number (*n*) of measurements and means in the intervention and control arm with 95 % confidence intervals (CI)

Visit	Intervention		Control		Adjusted difference
	*n*	Mean (95 % CI)	*n*	Mean (95 % CI)	difference (95 % CI)
3 weeks	318	−0.45 (−0.68 to −0.21)	286	−0.32 (−0.56 to −0.07)	−0.13 (−0.48 to 0.21)
6 weeks	346	−0.45 (−0.63 to −0.26)	316	−0.30 (−0.53 to −0.06)	−0.15 (−0.45 to 0.14)
12 weeks	369	−0.53 (−0.70 to −0.36)	324	−0.33 (−0.55 to −0.11)	−0.20 (−0.48 to 0.07)
24 weeks	363	−0.89 (−1.11 to −0.67)	328	−0.68 (−0.94 to −0.41)	−0.21 (−0.55 to 0.12)
2 years	331	−1.85 (−1.97 to −1.73)	312	−1.61 (−1.87 to −1.34)	−0.24 (−0.53 to 0.05)
5 years	242	−1.78 (−2.08 to −1.47)	221	−1.53 (−1.79 to −1.28)	−0.25 (−0.63 to 0.13)

The differences in means were calculated with linear regression adjusted for cluster design in addition to inverse-probability population weights

**Table 3:** Weight-for-age z-scores (WAZ) with number (*n*) of measurements and means in the intervention and control arm with 95 % confidence intervals (CI)

Visit	Intervention		Control		Adjusted difference
	*n*	Mean (95 % CI)	*n*	Mean (95 % CI)	difference (95 % CI)
3 weeks	320	−0.40 (−0.58 to −0.22)	289	−0.16 (−0.39 to 0.07)	−0.24 (−0.54 to 0.05)
6 weeks	345	−0.36 (−0.54 to −0.19)	314	−0.17 (−0.44 to 0.09)	−0.19 (−0.51 to 0.14)
12 weeks	366	−0.41 (−0.60 to −0.22)	324	−0.13 (−0.34 to 0.09)	−0.28 (−0.58 to 0.01)
24 weeks	364	−0.71 (−0.87 to −0.55)	329	−0.34 (−0.58 to −0.10)	−0.37 (−0.66 to −0.08)
2 years	331	−0.96 (−1.12 to −0.79)	312	−0.75 (−1.05 to −0.44)	−0.21 (−0.55 to 0.14)
5 years	235	−1.28 (−1.47 to −1.08)	219	−1.06 (−1.19 to −0.92)	−0.22 (−0.45 to 0.00)

The differences in means were calculated with linear regression adjusted for cluster in addition to inverse-probability population weights

Stunting was more common in the intervention than in the control arm with a significant difference at 2 years where 139 (45 %) were stunted in the intervention compared to 109 (37 %) in the control arm, odds ratio 1.7 (95 % confidence interval [CI] 1.1–2.4), Table 4. Wasting (WLZ<−2) was also more common in the intervention arm at the 12 and 24 week visits, but no clear differences were seen in the other interviews, Additional file 1: Table S3. Underweight (WAZ<−2) was more common in the intervention than in the control arm at the 5 years follow-up (OR 1.7 (1.0;2.8)). Adjusting odds ratios of wasting, stunting and underweight for socio-economic status in addition to cluster and site rendered similar results.

**Table 4:** Stunting and underweight in the intervention and control arms with odds ratios (OR) with 95 % confidence intervals (CI)

Visit	Stunting					Underweight				
	Intervention		Control		OR (95 % CI)	Intervention		Control		OR (95 % CI)
	*n*	%	*n*	%		*n*	%	*n*	%	
3 week	33	12	18	7	1.8 (0.83–4.0)	22	8	14	5	1.5 (0.69–3.4)
6 week	36	11	20	7	1.6 (0.88–3.0)	23	7	12	4	1.6 (0.69–3.6)
12 week	49	13	29	9	1.7 (0.85–3.2)	37	10	17	5	2.2 (0.99–4.7)
24 week	71	21	48	15	1.5 (0.92–2.4)	56	16	32	10	1.8 (0.87–3.7)
2 years	139	45	109	37	1.7 (1.1–2.4)	48	16	47	16	1.2 (0.58–2.3)
5 years	95	41	69	33	1.6 (0.90–2.7)	59	26	33	16	1.7 (1.0–2.8)

The odds ratios were calculated with a binomial generalised linear model adjusted for cluster and with inverse-probability population weights

A longitudinal assessment showed that the observed differences in WLZ, HAZ and WAZ were associated with the intervention, in that children whose mothers had received peer counselling had lower mean z-scores, Table 5 and Fig. 2. When the weight and length measurements at the 3 week visit were included in the model for HAZ, WAZ and WLZ, the adjusted differences were reduced. The longitudinal multilevel mixed-effect logistic regression model showed that arm allocation was associated with stunting, with an odds ratio (OR) of 1.7 (95 % CI 1.2 to 2.6), even after adjusting for HAZ at 3 weeks, 1.4 (95 % CI 1.2 to 1.9).

**Table 5:** Longitudinal assessment with mixed effects model adjusted differences in length/height-for-age z-scores (HAZ), weight-for-length z-score (WLZ) and weight-for-age z-score (WAZ) with differences in means in the intervention and control arm with 95 % confidence intervals (CI)

Measure	Analysis 1^a^	Analysis 2^b^
	mean (95 % CI)	mean (95 % CI)
HAZ	−0.21 (95 % CI −0.39 to −0.02)	−0.11 (95 % CI −0.24 to 0.01)
WLZ	−0.18 (95 % CI −0.34 to −0.02)	−0.01 (95 % CI −0.11 to 0.09)
WAZ	−0.26 (95 % CI −0.43 to −0.10)	−0.11 (95 % CI −0.22 to 0.00)

^a^Analysis 1: differences in means adjusted for cluster, site and child age

^b^Analysis 2: differences in means adjusted for cluster, site and child age and weight and length measurements at the 3 week

## Discussion

This study shows that the intervention with peer counselling to support 6 months of exclusive breastfeeding did not have positive growth outcomes. Both the intervention and the control arms performed poorly in terms of growth, with 45 % stunted children in the intervention arm and 37 % in the control arm at 2 years age and similarly at 5 years of age. In general, the intervention tended to have worse growth outcomes than the control arm. Research has shown that shifts in growth even within the normal range could be of importance [20].

A few other studies have investigated growth outcomes from interventions promoting exclusive breastfeeding. A randomised trial in Guinea Bissau found a small negative effect on ponderal growth from an intervention promoting exclusive breastfeeding [21]. Several other studies including MINIMat and PROBIT have not found any effect on growth [8, 9, 22–24]. Studies that have assessed breastfeeding in general compared to not breastfeeding have more positive findings on growth [25, 26]. In terms of other health outcomes, there has also been mixed results. The trial in Guinea Bissau did not find any other positive health outcomes similarly to the PROMISE-EBF trial which did also not find any impact on diarrhoea [4]. Some other studies have indicated short term positive effects on diarrhoea [21, 22, 24, 27].

It is important to recognise that even though the findings from this study do not show benefits from the peer counselling intervention – this does not imply that exclusive breastfeeding is harmful and there might be positive outcomes that we have not measured such as bonding between mother and child or immunological benefits. When including the feeding patterns in the model, the association between the intervention and stunting at 2 years of age was not altered, which is an argument supporting that the feeding pattern itself might not have been the key factor in this study. Thus, there might be other explanations for the findings. Even if mothers reported satisfaction with the peer counselling system and the peer counsellors were trained and supervised satisfactory [12, 15, 28], we lack information on duration, content and intensity on all the individual sessions.

One possible explanation is that the emphasis on the importance of giving only breast milk during the first 6 months might have reduced the recognition of the need to provide a sufficiently balanced complementary diet during the second half of infancy, or to start with supplements earlier when needed. The practice of exclusive breastfeeding might also have been prolonged beyond 6 months for some of the children. In fact, around 9 % of the children in the intervention arm breastfed exclusively at week 32 compared to none in the control arm, i.e. 1.5 months after it was recommended to start complementary feeding. Providing peer counselling with breastfeeding support that focus only on the first 6 months might thus be inadequate. Another possibility could be that the children in the intervention were to a lesser extent given additional liquids during severe or moderate diarrhoea. An important question is whether a 1-week course on breastfeeding promotion is enough to train peer-counsellors to give appropriate support.

When peer counsellors stressed the benefits of exclusive breastfeeding, this might also have given the families a false security that reduced health seeking behaviour. Proper health seeking behaviour might be essential in settings with a high burden of infectious diseases such as malaria and pneumonia. However, a study that was looking into vaccination in the same group did not find differences in timely vaccination between the intervention and control arms [29].

That some of the effect size was reduced by adjusting for length and weight at the 3 weeks visit, can either suggest that randomisation was not optimal, or that there was a difference that had emerged already between birth and the age of 3 weeks.

There are in fact some analogous examples to this study, including a large programme that was implemented in Benin, Mali and Ghana that aimed to improve child health [30]. The Accelerated Child Survival and Development programme put focus on nutritional counselling, vaccinations and antenatal care. Surprisingly, despite including these well-documented interventions, the programme did not show improved health outcomes in the intervention areas compared to the comparison areas.

This study has several strengths and limitations. Child growth was planned as one of the main outcomes. The field staff were well trained and regularly checked. There was low turnover among staff members. The standard deviations for the z-scores were just above one standard deviation, which indicate excellent measurement reproducibility. Randomisation of the intervention should have minimised confounding. The use of cluster randomisation might have increased the chance of having slight differences in baseline characteristics between the arm and control clusters compared to individual randomisation. There were some variations between the clusters in factors such as socio-economic status. A slightly higher proportion (non-significant) of the mothers in the intervention had lost a child previously. This could indicate an increased vulnerability of children in the intervention group. Still, adjusting for socio-economic factors did not have any effect on the findings. The cluster randomisation also reduced the potential contamination effect – when mothers talk with other mothers in their communities and potentially influence their behaviour – which is essential to do a proper assessment. Cluster adjustments were also taken into consideration in the models. Even though sample size calculation was not done for the growth outcomes, both the confidence intervals and post-hoc power calculations indicated relatively good analytical power. There was a substantial loss-to-follow-up for the last 5 year visit which could have introduced a selection bias. To check for sensitivity to the loss-to-follow-up and potential differences in baseline characteristics, regression using inverse-probability weighting were carried out. These results were similar to those without weighting indicating that substantial selection biases were unlikely. Traditional practices such as feeding the child with water supplements might have been done by family members without the mothers’ knowledge and thus difficult to account for, which could have reduced the differences in feeding patterns such as exclusive breastfeeding. Birth weight was often not available due to a large proportion delivering at home; however for nearly all children weight was measured 3 weeks after birth. There was a small and statistically non-significant difference in mean anthropometric measurements at 3 weeks between the arms. The intervention started with counselling during pregnancy and could have influenced growth at 3 weeks. Including growth measures at 3 weeks in the model slightly reduced the associations and some were then not statistically significant. As the absolute differences were relatively small even though the precision was relatively good, some of the association could be due to chance. To minimize potential multiple comparison problems as a number of analyses were conducted, the analyses were pre-planned prior to assessment of the data. Even though the data collectors were not made aware of the arm allocation, it is possible that mothers who took part in the intervention mentioned this directly or indirectly during the interviews, and thereby somehow influenced the anthropometrists. However, if such bias was present, it would probably have had the opposite effect of what was observed. Considering the consistencies and trends between all the different measurements in all the interviews, it is unlikely that all the negative effects from the intervention on growth are due to chance alone.

## Conclusion

There were no growth advantages of this community-based intervention that promoted exclusive breastfeeding for 6 months with peer-counsellors. While stunting was widespread at 2 and 5 years of age in both arms, it was more common in the intervention arm.

It is questionable whether community-based support from lay people with short training and focussing only on exclusive breastfeeding, leaving aside aspects such as introduction of complementary feeds, is an appropriate strategy to improve child health and development.

## Abbreviations

CI, confidence interval; HAZ, length/height-for-age z-score; IQR, inter-quartile range; OR, odds ratio; WAZ, weight-for-age z-score; WHO, World Health Organisation; WLZ, weight-for-length z-score

## Additional file

Additional file 1: Table S1. Background characteristics of the populations of the 5 years follow-up and those lost-to-follow-up at 5-years visit. **Table S2.** Weight-for-length/height z-scores (WLZ) with number (n) of measurements and means in the intervention and control arm with 95 % confidence intervals (CI). The differences in means are adjusted for cluster in addition to inverse-probability population weights. **Table S3.** Wasting (WLZ<−2) in the intervention and control arms with odds ratios (OR) with 95 % confidence intervals (CI). The odds ratios are adjusted for cluster and with inverse-probability population weights. **Figure S1 (A,B,C,D).** Distribution of length/height-for-age (HAZ) and weight-for-length z-scores (WLZ) at 3 weeks (A), 24 weeks (B) and 2 years (C) of age presented with histograms. Similarly at 5 years, the distribution of heigth-for-age (HAZ) and weight-for-age z-scores (WAZ) is shown (D). The proportion below the red dotted vertical lines represent stunted (histograms on the left side) and wasted children (histograms on right hand side). The blue dotted vertical lines represent the mean of the WHO growth standard. (PDF 300 kb)
