# Supplementary material for: Effects of an exclusive breastfeeding intervention for six months on growth patterns of 4–5 year old children in Uganda: the cluster-randomised PROMISE EBF trial
Source: BMC Public Health. 2016 Jul 12;16:555. doi: 10.1186/s12889-016-3234-3 (PMC4942943; doi:10.1186/s12889-016-3234-3)
Supplement: Additional file 1: Table S1. — Background characteristics of the populations of the 5 years follow-up and those lost-to-follow-up at 5-years visit. Table S2. Weight-for-length/height z-scores (WLZ) with number (n) of measurements and means in the intervention and control arm with 95 % confidence intervals (CI). The differences in means are adjusted for cluster in addition to inverse-probability population weights. Table S3. Wasting (WLZ<−2) in the intervention and control arms with odds ratios (OR) with 95 % confidence intervals (CI). The odds ratios are adjusted for cluster and with inverse-probability population weights. Figure S1 (A,B,C,D). Distribution of length/height-for-age (HAZ) and weight-for-length z-scores (WLZ) at 3 weeks (A), 24 weeks (B) and 2 years (C) of age presented with histograms. Similarly at 5 years, the distribution of heigth-for-age (HAZ) and weight-for-age z-scores (WAZ) is shown (D). The proportion below the red dotted vertical lines represent stunted (histograms on the left side) and wasted children (histograms on right hand side). The blue dotted vertical lines represent the mean of the WHO growth standard. (PDF 300 kb) [file 12889_2016_3234_MOESM1_ESM.pdf]

Table S1: Background characteristics of the populations of the 5 years follow-up and those lost-to-follow-up at 5-years visit.

**Visit**	**5 years visit**				**Lost-to-follow-up at 5-years visit**			
	**Control**		**Intervention**		**Control**		**Intervention**	
**Categorical data**	n	%	n	%	n	%	n	%
Eligible mother-infant pairs	221		242		148		154	
Multipara (child has siblings)	174	79	196	82	99	67	110	71
Previous child death	56	32	73	37	24	16	36	23
Female child	110	50	119	49	71	48	75	49
Mother married or cohabiting	209	95	224	93	129	87	139	90
Living in rural area	172	78	187	77	104	70	103	67
Electricity in house	37	17	30	13	33	22	23	15

**Continuous data**	median	IQR	median	IQR	median	IQR	median	IQR
Maternal age	25	(21–31)	26	(21–30)	23	(20–28)	24	(20–28)
Maternal education	6	(4–9)	6	(4–7)	7	(5–9)	6	(4–8)
Child age	53	(49–61)	55	(50–62)	unknown		unknown	
Maternal body mass index	22	(21–24)	22	(20–24)	22	(20–23)	21	(20–23)
Socio-economic quintile	3	(2–4)	3	(2–4)	4	(2–5)	3	(2–4)

Table S2: Weight-for-length/height z-scores (WLZ) with number (n) of measurements and means in the intervention and control arm with 95% confidence intervals (CI). The differences in means are adjusted for cluster in addition to inverse-probability population weights.

Visit	Intervention		Control		Adjusted difference
	n	mean (95 %CI)	n	mean (95 %CI)	difference (95 %CI)
3 weeks	315	-0·19 (-0·35 to -0·03)	286	-0·01 (-0·21 to 0·19)	-0·18 (-0·43 to 0·07)
6 weeks	343	0·14 (-0·01 to 0·30)	313	0·20 (0·04 to 0·36)	-0·06 (-0·27 to 0·14)
12 weeks	366	0·09 (-0·08 to 0·26)	325	0·27 (0·12 to 0·42)	-0·18 (-0·40 to 0·04)
24 weeks	363	-0·09 (-0·24 to 0·06)	328	0·21 (0·05 to 0·38)	-0·30 (-0·52 to -0·08)
2 years	331	-0·06 (-0·21 to 0·10)	312	0·10 (-0·18 to 0·37)	-0·15 (-0·44 to 0·15)

* WLZ scores are not calculated by WHO Anthro+ for children above the age of 5 years (thus z-scores from the last visit are excluded from the presentation)

Table S3: Wasting (WLZ<-2) in the intervention and control arms with odds ratios (OR) with 95% confidence intervals (CI). The odds ratios are adjusted for cluster and with inverse-probability population weights.

Visit	Intervention		Control		OR (95% CI)
	n	%	n	%	
3 week	15	5	14	5	0·90 (0·42–1·9)
6 week	12	4	8	3	1·4 (0·62–3·1)
12 week	22	6	10	3	2·1 (1·0–4·3)
24 week	26	8	10	3	2·5 (1·1–5·7)
2 years	16	5	16	5	1·1 (0·51–2·5)

* WLZ scores are not calculated by WHO Anthro+ for children above the age of 5 years (thus z-scores from the last visit are excluded from the presentation)

FigureS1A

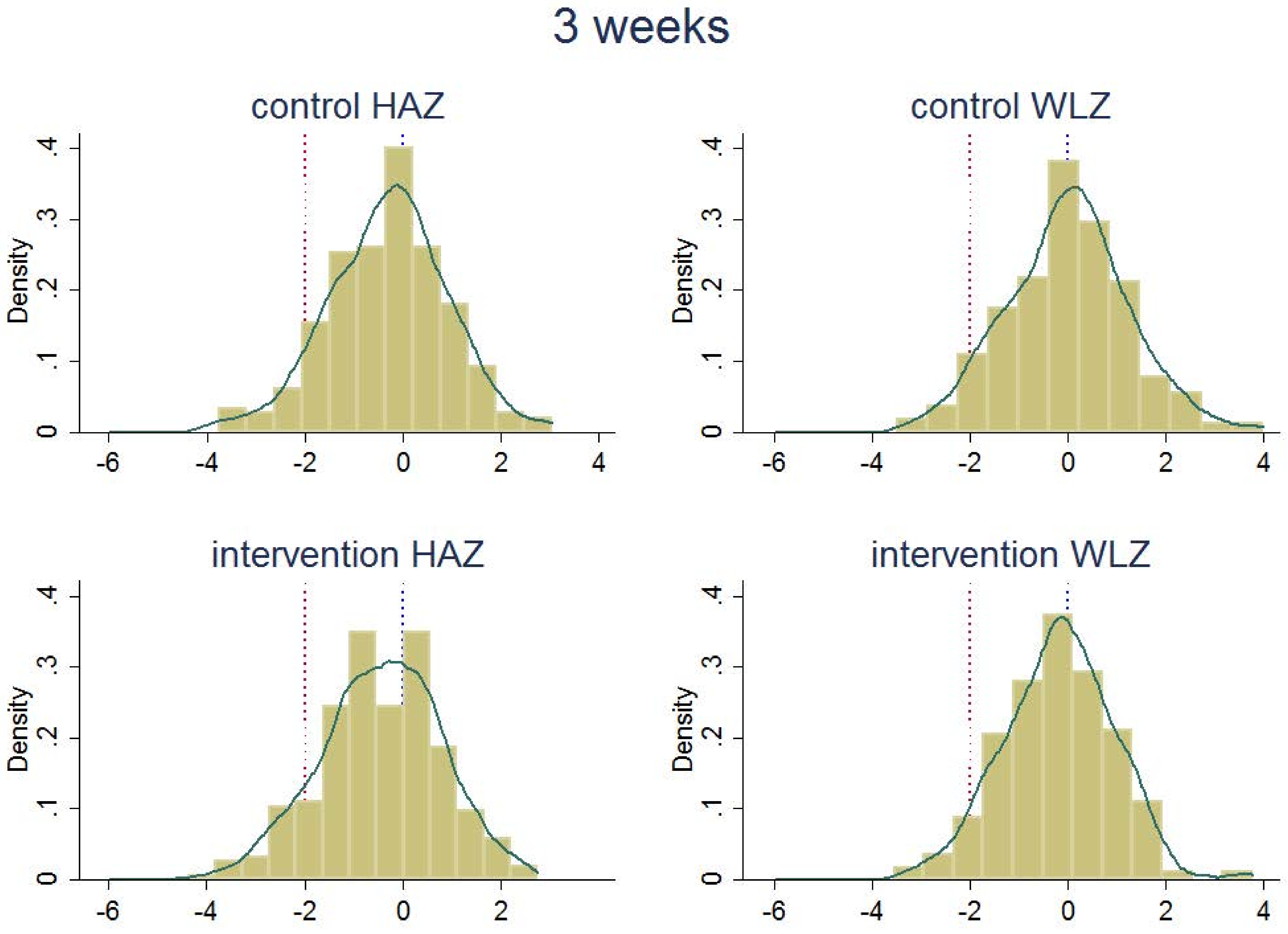

3 weeks
control HAZ
control WLZ
intervention HAZ
intervention WLZ
Density
0
.1
.2
.3
.4
-6
-4
-2
0
2
4

FigureS1B

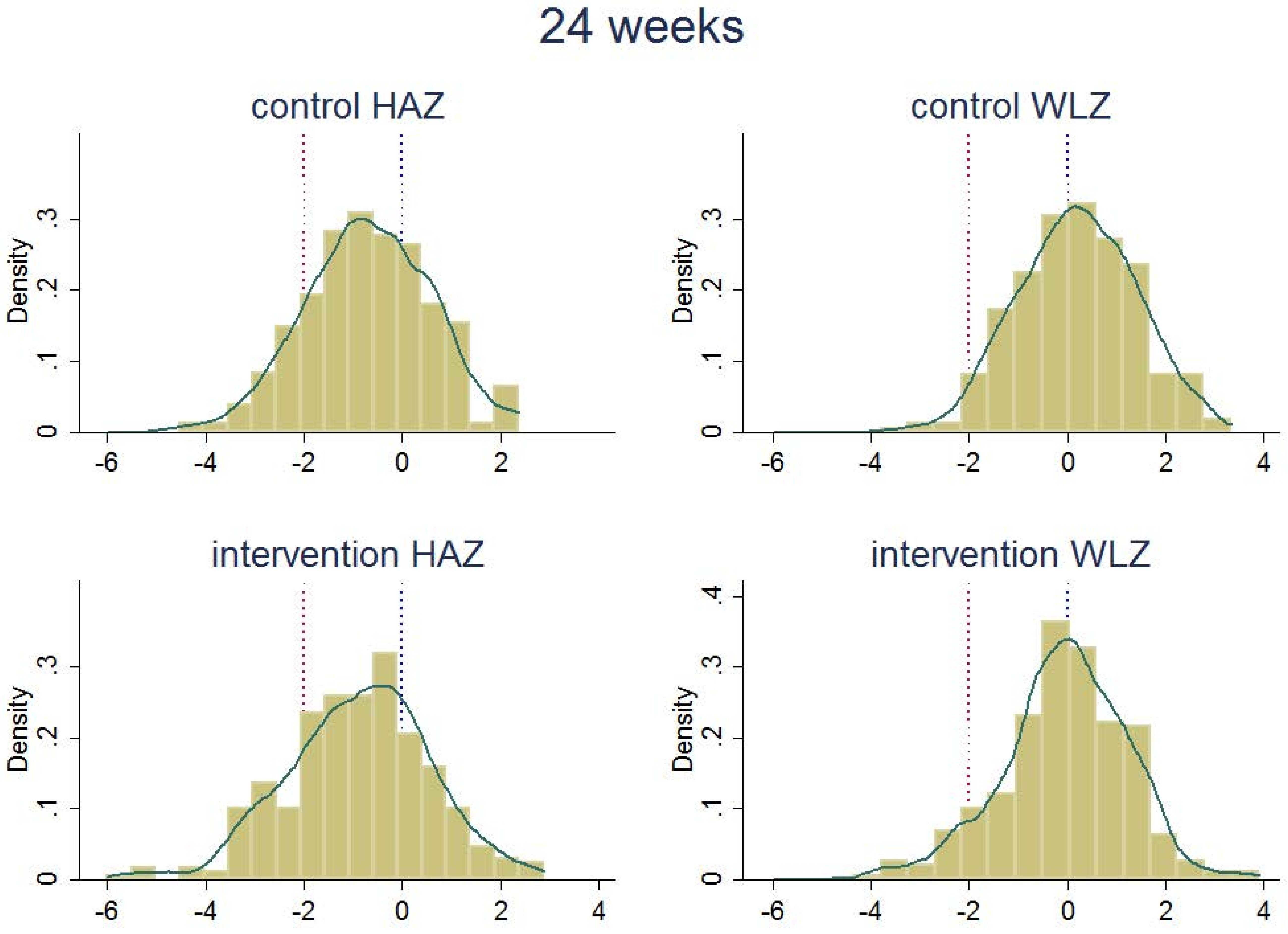
24 weeks
control HAZ
control WLZ
intervention HAZ
intervention WLZ
Density
0
.1
.2
.3
.4
-6
-4
-2
0
2
4

FigureS1C

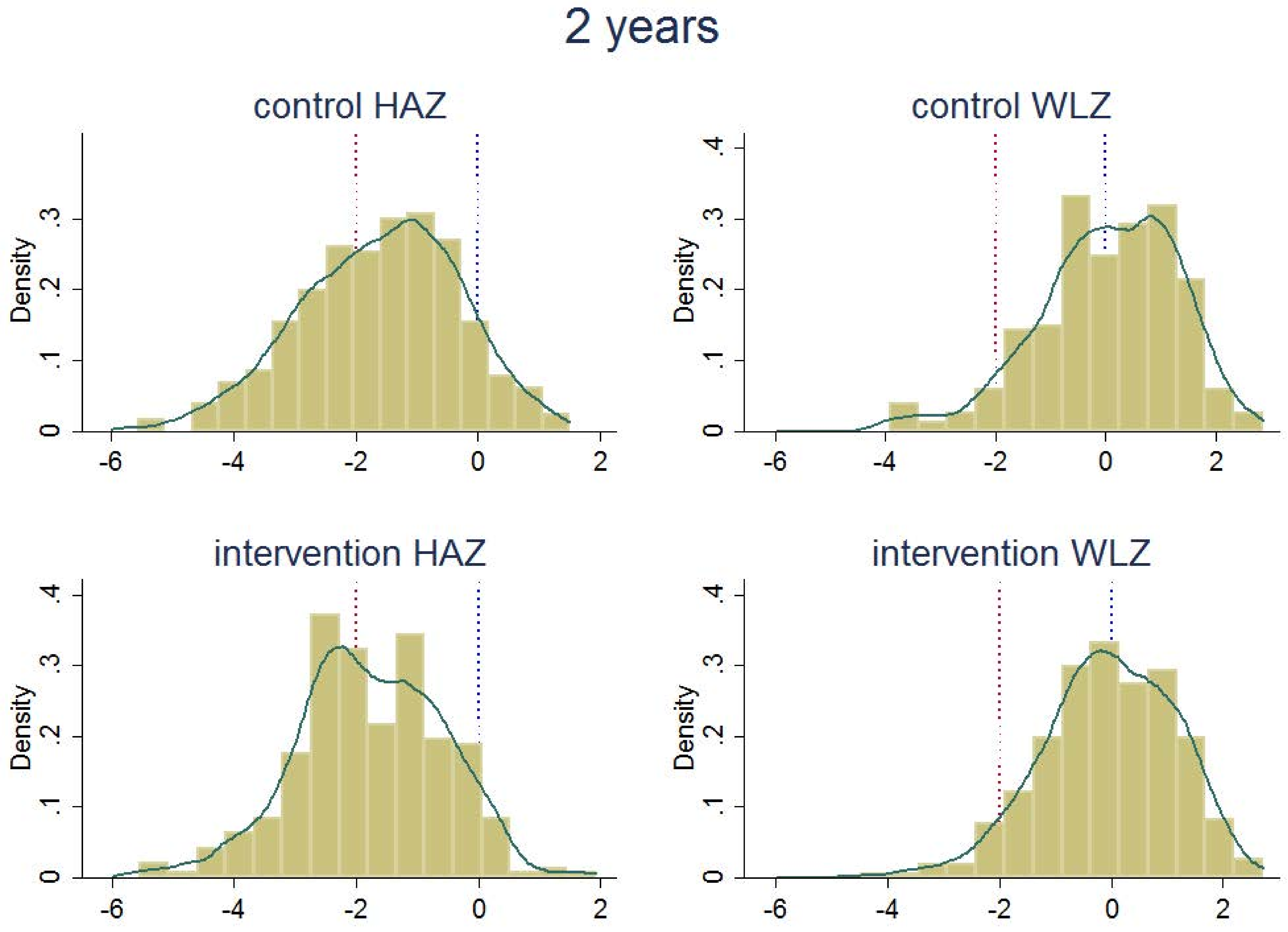

2 years
control HAZ
control WLZ
intervention HAZ
intervention WLZ
Density
0
.1
.2
.3
.4
-6
-4
-2
0
2

FigureS1D

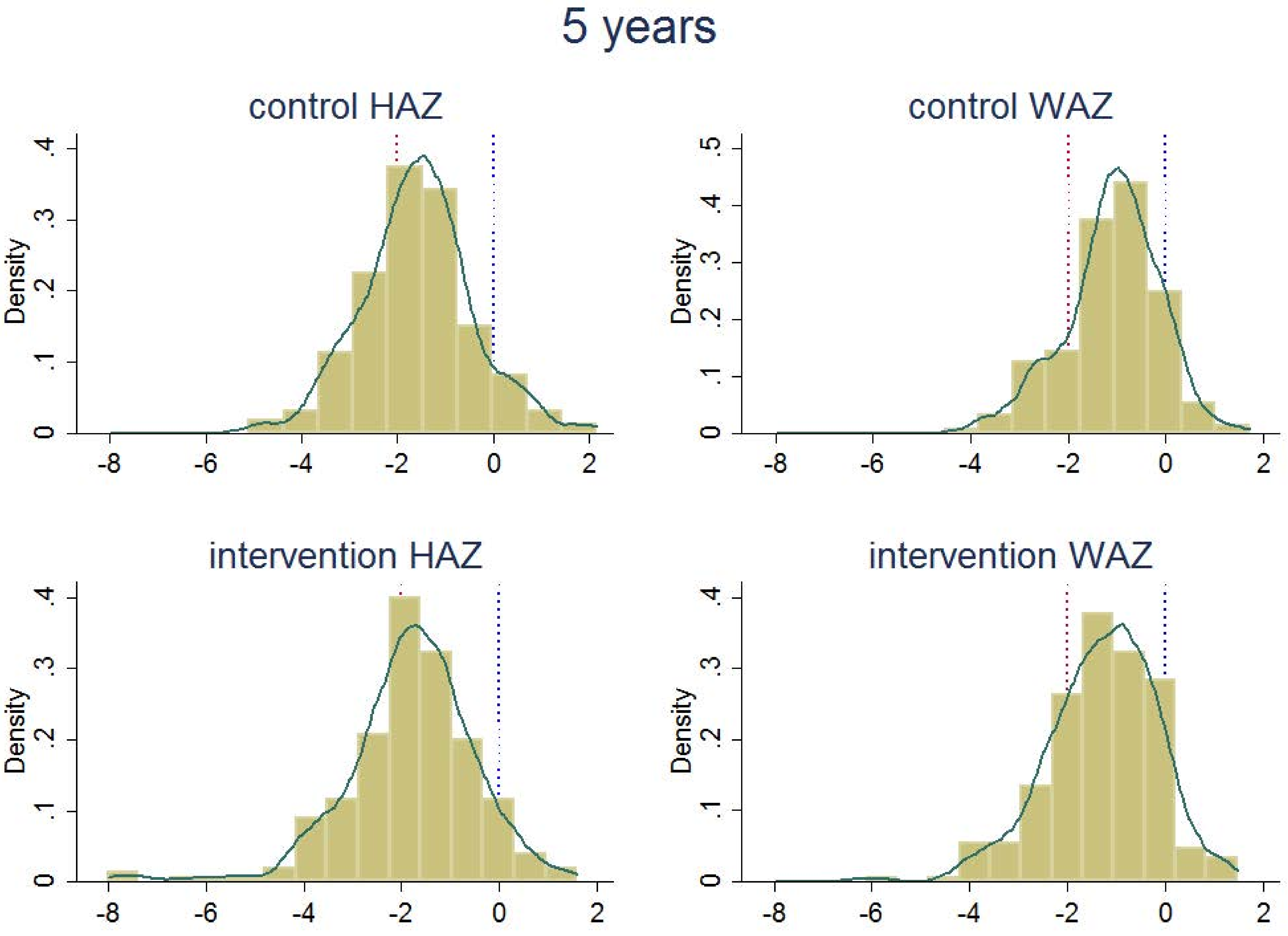
5 years
control HAZ
control WAZ
intervention HAZ
intervention WAZ
Density
0
.1
.2
.3
.4
.5
-8
-6
-4
-2
0
2
